# Real-time multiple cross displacement amplification assay for rapid and sensitive detection of Haemophilus influenzae

**DOI:** 10.3389/fcimb.2022.1004183

**Published:** 2022-09-27

**Authors:** Chunrong Sun, Nan Jia, Xiaolan Huang, Fei Xiao, Juan Zhou, Yu Zhang, Jin Fu, Zheng Xu, Dong Qu, Xiaodai Cui, Yi Wang

**Affiliations:** ^1^ Experiment Center, Capitital Institute of Pediatrics, Beijing, China; ^2^ Department of Critical Medicine, Children’s Hospital Affiliated to the Capital Institute of Pediatrics, Beijing, China

**Keywords:** Haemophilus influenzae, multiple cross displacement amplification, rapid diagnosis, real-time, loop mediated isothermal amplification, PCR

## Abstract

Haemophilus influenzae is an opportunistic pathogen usually causing bacteremia, meningitis, and pneumonia in children. Here, we developed a method based on multiple cross displacement amplification (MCDA) method and real-tme fluorescence technique for rapid detection of H. influenzae. A set of 10 primers was designed for the H. influenzae real-time MCDA reaction, and a core primer was modified with a restriction endonuclease recognition sequence, a fluorescent, and a quencher according to the principle of the real-time MCDA assay. The H. influenzae real-time MCDA reactions were performed using a fluorescence instrument at 63°C for 40 min. The H. influenzae real-time MCDA assay can specifically detect H. influenzae without any cross-reaction with other bacteria as our results confirmed. The sensitivity of our assay is as low as 10 CFU per reaction. To validate its feasibility, our assay was applied to 40 DNA extracted from sputum samples. The results obtained from H. influenzae real-time MCDA were compared with that of H. influenzae–loop-mediated isothermal amplification (H. influenzae-LAMP) and MCDA-based lateral flow biosensor (MCDA-LFB). The positive rate of the real-time MCDA assay was 62.5%, which was consistent with the H. influenzae-MCDA-LFB assay, but was more sensitive than H. influenzae-LAMP (57.5%). Furthermore, the H. influenzae real-time MCDA assay takes only 40 min, which was less than that of a traditional PCR test. Taken together, the H. influenzae real-time MCDA assay reported here offers a new and valuable diagnostic tool for the reliable and rapid detection of H. influenzae.

## Introduction

Haemophilus influenzae (H. influenzae) was a Gram-negative bacterium isolated primarily from the human respiratory tract. As an opportunistic pathogen, H. influenzae was one of the most common causes of bacterial pneumonia, which was the largest infectious cause of death in children worldwide and accounted for 14% of deaths of children under 5 years old (2021; Marrs et al., 2001; O’Brien et al., 2019). Moreover, H. influenzae could infect various parts of the human body, causing diseases such as meningitis, epiglottis, and septic arthritis (Marrs et al., 2001; Shooraj et al., 2019; Fu et al., 2021). Children aged from 2 months to 5 years old were more susceptible to H. influenzae infections because of the incapacity of transplacental maternal antibodies to the newborns and the lack of “natural antibodies” for the older children and the adults (Ladhani et al., 2012; Dong et al., 2020).

Current laboratory diagnostics of H. influenzae infection mainly consist of culture, antigen tests, polymerase chain reaction (PCR) assays, and isothermal nucleic acid amplification technique. Besides being time-consuming and labor-intensive, culture results were easily influenced by the prior antibiotic usage, and the conditions of the specimens’ acquirement, transport, and storage. The results of antigen test (slide agglutination serotyping) were prone to have inconsistencies (LaClaire et al., 2003). The PCR-based method provided a valuable approach for the detection of H. influenzae and definitely improved the specificity and sensitivity for the laboratory diagnosis of H. influenzae infections (Marty et al., 2004; Tian et al., 2012; Gadsby et al., 2016; Fan et al., 2018; Albuquerque et al., 2019; Watts and Holt, 2019). However, in the last 20 years, novel isothermal amplification technologies, which could specifically accumulate target sequences under isothermal conditions, such as loop-mediated isothermal amplification (LAMP) and multiple cross displacement amplification (MCDA) assays, had been widely reported. In spite of getting over the limitation of thermal cycles, the MCDA technique, above all, is more superior in terms of analytical specificity, sensitivity, and reliability by using 10 primers (2 displacement primers, 2 cross primers, and 6 amplification primers) to recognize the target sequences instead of 2 in PCR or 6 in LAMP. The isothermal amplification assays were very much recommended for the resource-limited laboratory settings in the previous reports for its simple requirements of equipment and permitted use (Wang et al., 2015; Wang et al., 2016; Wang et al., 2019; Li et al., 2019; Li et al., 2020; Soroka et al., 2021). The determination of the results using visual detection reagent (VDR) or lateral flow biosensor (LFB), however, was not completely objective, and might suffer from false-positive results caused by primer dimers or product-induced pollution when using the LFB test.

In the last 2 years, on account of the global epidemic of COVID-19, increasingly more modern and expensive instruments and devices are equipped to satisfy the huge demand of nucleic acid detection even in the primary medical institutions. Here, we devised a real-time MCDA assay that integrated MCDA technique with restriction endonuclease and real-time fluorescence examination method. This assay was carried out by using a fluorescence quantitative PCR instrument, and the results were real-time monitored, avoiding subjective error or pollution caused by using VDR or the LFB test. In addition, this assay was further validated by comparing with the results acquired by the MCDA-LFB assay and the LAMP test on the examination of clinical samples.

## Materials and methods

### Reagents and instruments

DNA Isothermal Amplification Kit, restriction endonuclease (Nb.BsrDI), visual detection reagent (VDR), biotin-dCTP, and nanoparticle-based lateral flow biosensor (LFB) were obtained from Huidexin Biotech Co., Ltd. (Tianjin, China). Primers and labeled primers used in this study were synthesized by AoKe Biotech Co., Ltd (Beijing, China). DNA extraction and purification kits were purchased from TransGen Biotech Co., Ltd (Beijing, China). Real-time turbidimeter LA-320C was purchased from Eiken Chemical Co., Ltd, Japan. Fluorescence quantitative PCR instrument ABI 7500 was purchased from Applied Biosystem Inc., USA.

### Primer design

**Figure 1:**
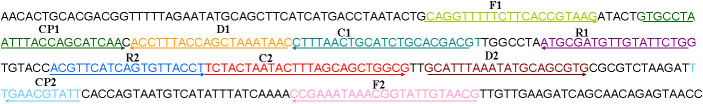
The location of the primer sequences used in this study on the targeting outer membrane protein (OMP) P6 gene of H. influenzae. Right and left arrows show sense and complementary sequences, respectively. The colored text indicates the position of primers, including two displacement primers (F1 and F2), two cross primers (CP1 and CP2), and six amplification primers (C1, C2, D1, D2, R1, and R2).

A set of 10 primers targeting the outer membrane protein (OMP) P6 gene (GenBank no. L42023.1) of H. influenzae was designed using primer premier 5.0. The primers included two displacement primers (F1 and F2), two cross primers (CP1 and CP2), and six amplification primers (C1, C2, D1, D2, R1, and R2). The sequences, locations, and the modification of the primers are shown in **Table 1** and **Figure 1**. The Black Hole Quencher 1 (BHQ1) and the fluorophore FAM were used in the real-time MCDA reaction for real-time detection.

**Table 1:** The primers used in the study.

Primer name	Sequence and modifications	Length
P6-F1	CAGGTTTTTCTTCACCGTAAG	21
P6-F2	CGTTACAATACCGTTTATTTCGG	23
P6-CP1	ACGTCGTGCAGATGCAGTTAAAG-GTGCCTAATTTACCAGCATCAA	46
P6-CP2	TCTACTAATACTTTAGCAGCTGGCG-CATTACTGGTGAATACGTTCAA	48
P6-C1	ACGTCGTGCAGATGCAGTTAAAG	23
P6-C2	TCTACTAATACTTTAGCAGCTGGCG	25
P6-D1	GTTATTTAGCTGGTAAAGGT	20
P6-D2	GCATTTAAATATGCAGCGTG	20
P6-R1	CCAGAATACAACATCGCAT	19
P6-R2	ACGTTCATCAGTGTTACCT	19
P6-RT-D1*	5’-FAM-TGCAATG-GTT-(BHQ1)-ATTTAGCTGGTAAAGGT-3’	23
P6-D1#	5’-FAM-GTTATTTAGCTGGTAAAGGT-3’	

*Labeled primer used for real-time MCDA assay.

^#^Labeled primer used for the MCDA-LFB assay.

### MCDA reaction and the LFB test

The volume of the MCDA reaction system was 25 μl, containing 12.5 μl of 2 × isothermal reaction buffer, 0.4 μM each of displacement primers (F1 and F2), 1.6 μM each of cross primers (CP1 and CP2), 0.8 μM each of amplification primers [C1, C2, D1#(Fam labeled), D2, R1, and R2], 1.0 μl of Bst 2.0 DNA polymerase (8U), 0.5 μl of biotin-14-dCTP, 1.2 μl of VDR, and 1 μl of template for pure culture or 5 μl for clinical samples. The reactions were conducted at 63°C for 1 h using a turbidimeter or a traditional PCR instrument. The results were monitored by a real-time turbidimeter or directly observed by the color of VDR, and further validated by the LFB test. To indicate the MCDA results, 5 µl of MCDA reaction products was added to the sample pad of the LFB, followed with 100 μl of running buffer (10 mM PBS, pH 7.4, with 1% Tween 20). The results were reported within 2 min, with two red lines at both TL and CL representing a positive result and only one red line at CL meaning negative.

### The optimal temperature for the H. influenzae real-time MCDA reaction

The reaction temperatures for the H. influenzae MCDA reaction were optimized by conducting the reaction at 60–67°C with 1°C intervals. DNA templates of H. influenzae strains were used as positive control and distilled water (DW) was used as blank control. The amplification process was real-time monitored by a turbidimeter for 1 h and then incubated at 85°C for 5 min to stop the reaction. The obtained optimal temperature was subsequently used for the real-time MCDA assay.

### The standard real-time MCDA reaction

To further evaluate the feasibility of the primers, the real-time MCDA assay was conducted in a volume of 25-μl reaction system containing 12.5 μl of 2 × isothermal reaction buffer, 0.4 μM each of displacement primers (F1 and F2), 1.6 μM each of cross primers (CP1 and CP2), 0.8 μM each of amplification primers [C1, C2, D1*(restriction endonuclease recognition sequence, FAM and BHQ1 labeled), D2, R1, and R2], 1.0 μl of Bst 2.0 DNA polymerase (8U), 1.0 μl of Nb.BsrDI restriction endonuclease, and 1 μl of template for pure culture (5 μl for clinical sample). The reaction was conducted on RT-PCR instruments for 40 cycles with the setting mode of 30 s at 63°C followed by another 30 s at 63°C. In the real-time MCDA reaction system containing positive templates, double-stranded products terminated with the restriction endonuclease recognition site and its complementary sequences will be generated by Bst polymerase. Then, the double-stranded terminal sequences will be digested by restriction endonuclease, resulting in the release of fluorescence signals.

### Sensitivity and repeatability of the H. influenzae real-time MCDA assay

In order to get the detection limit of the H. influenzae real-time MCDA assay, cultured H. influenzae were serially diluted (10^-1^–10^-9^), 100 μl of each dilution was spread on the chocolate blood agar in triplicate, and the colony-forming units (CFU) of the appropriate dilution were counted after incubation in a 5%–10% carbon dioxide incubator at 37°C for 24 h. Then, the appropriate dilutions were serially adjusted to 2×10^8^, 2×10^7^, 2×10^6^, 2×10^5^, 2×10^4^, 2×10^3^, 2×10^2^, and 2×10^1^ (CFU/ml). DNA templates (100 μl) were extracted from 500 μl of each adjusted dilution for the following analysis of the limit of detection (LoD) of real-time MCDA and MCDA-LFB assay. Accordingly, 1 μl of each of the serial templates was added into the reaction systems to determine the LoD of the assays. The LoD template amounts were repeatedly tested twice.

### Specificity of the H. influenzae real-time MCDA assay

In order to evaluate the specificity of the real-time MCDA assay, DNA templates from 2 H. influenzae strains and 28 non-H. influenzae strains (**Table 2**) were tested by the real-time MCDA assay, and each sample was tested at least twice.

**Table 2:** Bacterial strains used to determine the specificity of LAMP.

Bacteria	Strain no.(source of strains) a	No. of strains	H. influenza-real-time MCDA b
Haemophilus influenzae	Isolated strains (CIP)	2	P
Enteroinvasive Escherichia coli	Isolated strains (CDC)	1	N
Enteroadherent Escherichia coli	Isolated strains (CDC)	1	N
Enterotoxic Escherichia coli	Isolated strains (CDC)	1	N
Enteropathogenic Escherichia coli	Isolated strains (CDC)	1	N
Shiga toxin-producing Escherichia coli	Isolated strains (CDC)	1	N
Streptococcus suis	Isolated strains (CDC)	2	N
Listeria innocua	Isolated strains (CDC)	1	N
Klebsiella pneumoniae	Isolated strains (CDC)	3	N
Streptococcus salivarius	Isolated strains (CDC)	1	N
Mycobacterium tuberculosis	Isolated strains (CDC)	1	N
Corynebacterium striatum	Isolated strains(CDC)	1	N
Nocardia asteroides	Isolated strains (CDC)	1	N
Moraxella catarrhalis	Isolated strains (CDC)	1	N
Stenotrophomonas maltophilia	Isolated strains (CDC)	1	N
Staphylococcus epidermidis	Isolated strains (CDC)	1	N
Staphylococcus amber	Isolated strains (CDC)	1	N
Staphylococcus haemolyticus	Isolated strains (CDC)	1	N
Neisseria meningitidis	Isolated strains (CDC)	1	N
Streptococcus pneumoniae	Isolated strains (CDC)	1	N
Streptococcus pyogenes	Isolated strains (CDC)	1	N
Pseudomonas aeruginosa	Isolated strains (CDC)	1	N
Salmonella	Isolated strains (CDC)	1	N
Shigella sonnei	Isolated strains (CDC)	1	N
Shigella baumannii	Isolated strains (CDC)	1	N
Enterococcus faecalis	Isolated strains (CDC)	1	N

a CIP, Capital Institute of Pediatrics. CDC, Chinese Center for Disease Control and prevention.

b P, positive; N, negative. Only H. influenzae could be detected by H. influenza-real-time MCDA assay, indicating the extremely high selectivity of the method.

### Clinical application of the H. influenzae real-time MCDA assay

For evaluation of the clinical feasibility, DNA isolates from 40 sputum specimens (23 H. influenzae-LAMP test positive and 17 negative) were retrospectively tested using the H. influenzae real-time MCDA assay. The sputum specimens were collected from the hospitalized children in the Children’s Hospital affiliated to Capital Institute of Pediatrics, Beijing, China. All the samples were obtained with informed consent signed by the guardians of the participants.

### Statistical analysis

Comparison between the two methods, LAMP and real-time MCDA assay, was analyzed by the χ2 test with the SPSS software (version 11.5), and p < 0.05 was considered statistically significant.

## Results

### The effectiveness of the primer set for H. influenzae

**Figure 2:**
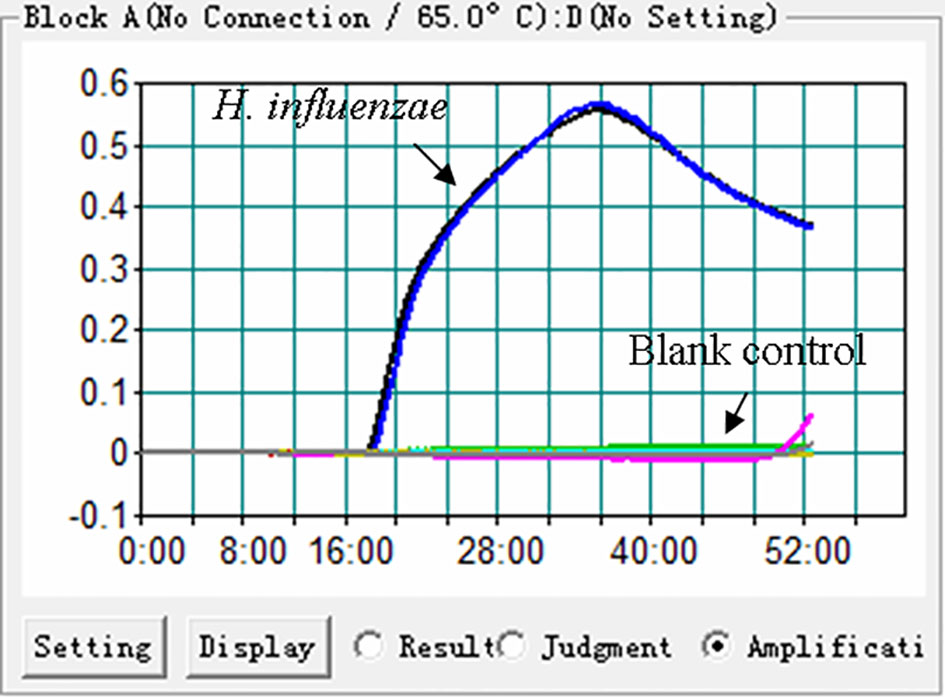
Effectiveness of the primer set for the H. influenzae-MCDA reaction. The DNA templates extracted from H. influenzae strains were effectively amplified with MCDA reaction at 65°C, and there is no reaction for the blank controls (DW).

In order to confirm the effectiveness of the primer set (**Figure 1**, **Table 1**) targeting the P6 gene of H. influenzae, the MCDA reactions were conducted at 65°C for 1 h using DNA templates extracted from H. influenzae strains. The reaction process was recorded through real-time measurement of turbidity. As shown in **Figure 2**, the templates of H. influenzae strains were effectively amplified and no reaction was observed from DW. Thus, the primer set was suitable to establish the H. influenzae real-time MCDA assay.

### The optimal temperature for the H. influenzae real-time MCDA assay

**Figure 3:**
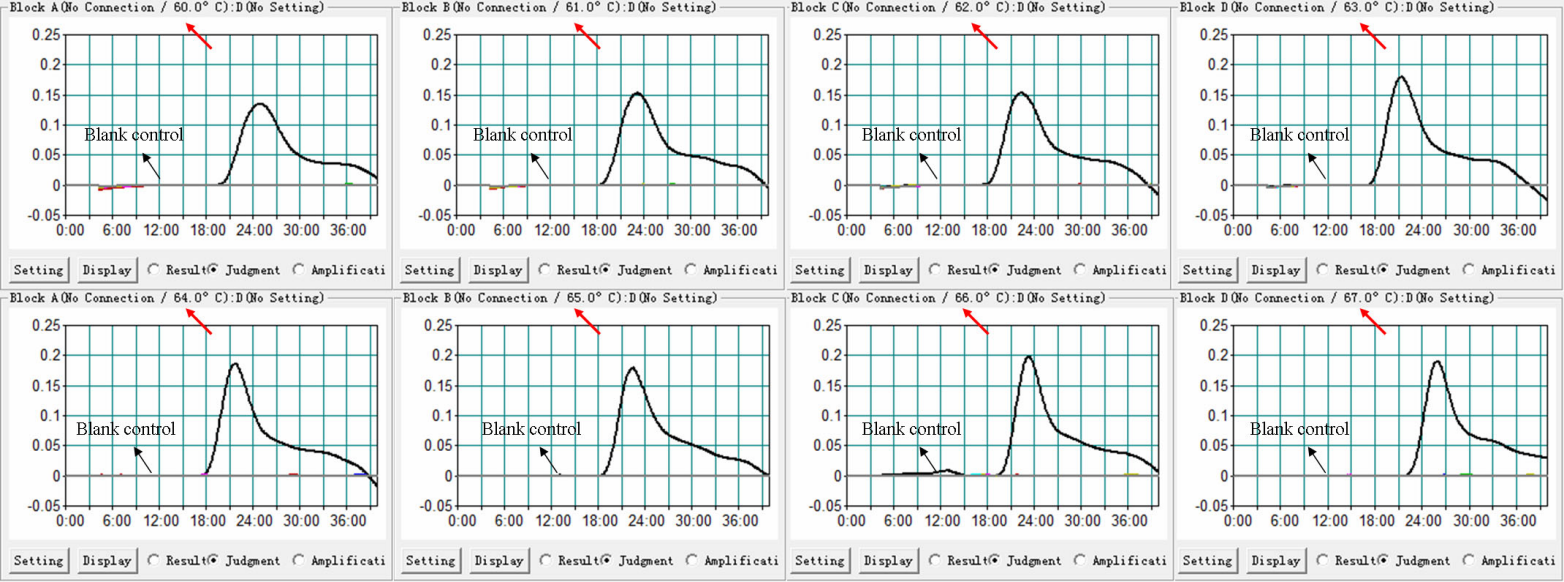
Temperature optimization for H. influenzae-real-time MCDA assay. MCDA reactions detecting H. influenzae were conducted using real-time turbidimeter, and the kinetic curves at different temperatures ranging from 60 to 67°C were acquired, showing that 63°C was the optimal temperature.

As shown in **Figure 3**, temperatures ranging from 60°C to 67°C with 1°C intervals were employed to conduct the real-time MCDA reactions for optimization of the reaction temperature. It was concluded that 63°C is the optimal reaction temperature for the primer set, since the fastest peak was obtained under this condition. Then, 63°C was subsequently used for the real-time MCDA assay as the optimal temperature.

### Sensitivity of the H. influenzae real-time MCDA assay

**Figure 4:**
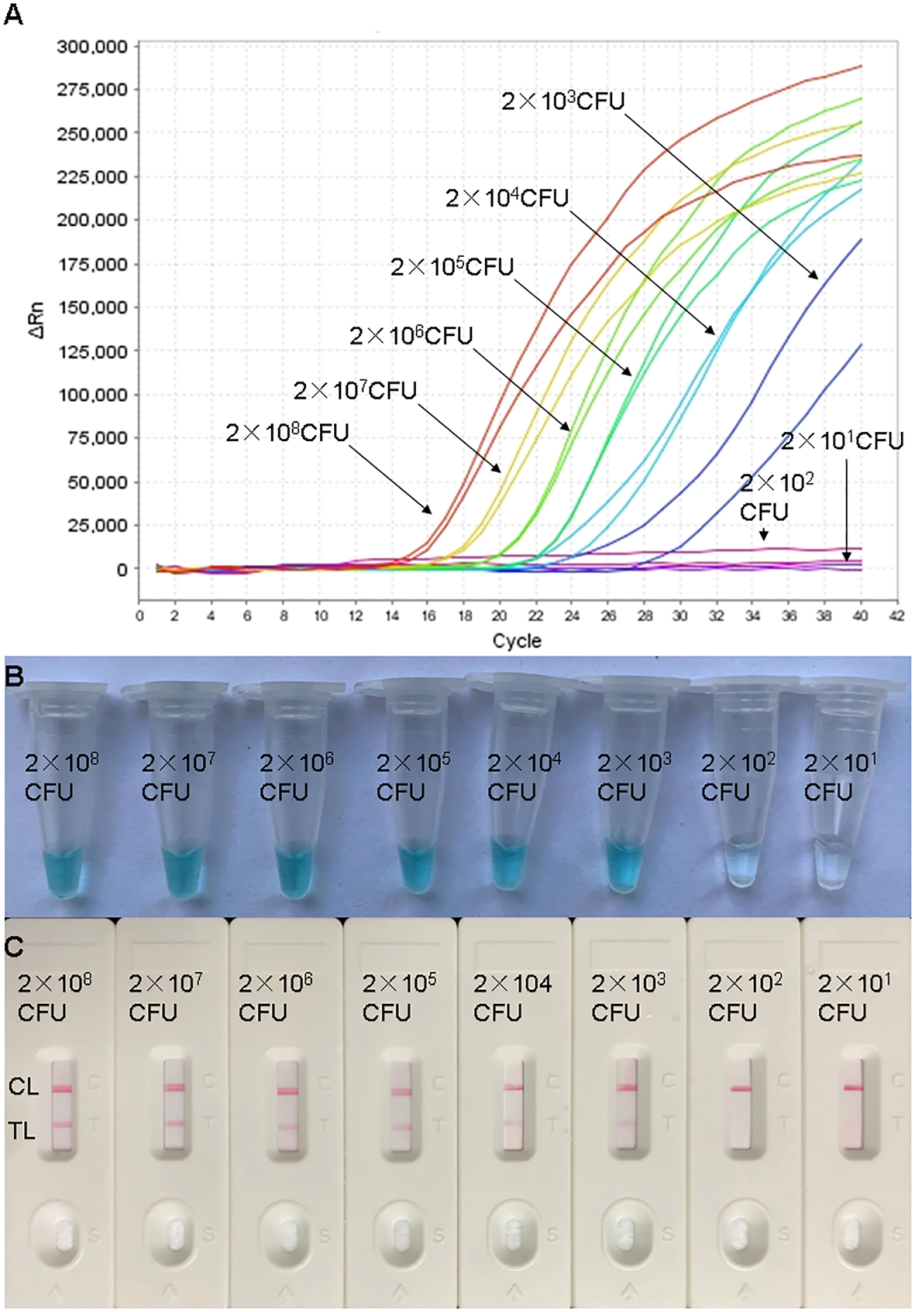
Sensitivity confirmation of H. influenzae-real-time MCDA assay. Sensitivity of the H. influenzae-real-time MCDA assay was analyzed by using continuous dilutions from 2×10^8^ to 2×10^1^ CFU/ml. The real-time MCDA reactions with various levels of DNA templates were repeatedly tested twice. Signals **(A)** representing DNA levels showed that the LoD of the H. influenzae-real-time MCDA was 10 CFU per tube. The sensitivity was also confirmed with indication of visual detection reagent **(B)** and LFB test **(C)** for the production of MCDA reactions. **(C)** TL, test line; CL, control line.

In order to assess the sensitivity of the real-time MCDA assay, the processes were carried out using DNA templates extracted from adjusted dilutions of cultured H. influenzae. The positive reactions were observed in about 15 min. The LoD of the real-time MCDA assay for the detection H. influenzae was 2×103 CFU/ml (~10 CFU/tube, **Figure 4A**), which was in complete accordance with the MCDA-LFB assay (**Figures 4B, C**).

### Specificity of the H. influenzae real-time MCDA assay

**Figure 5:**
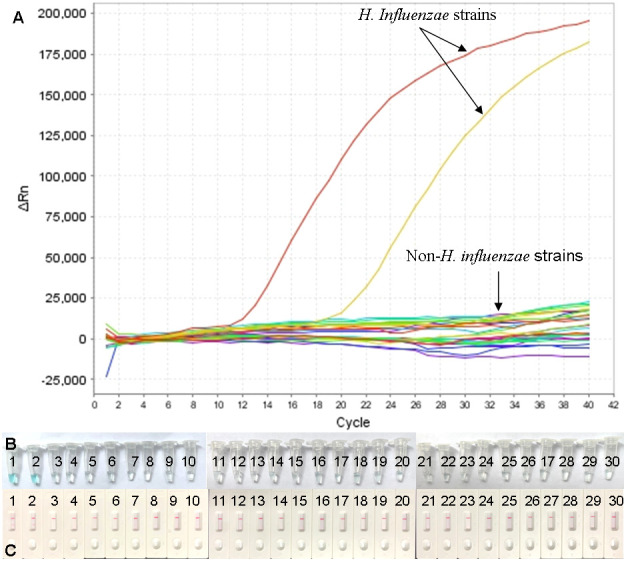
Specificity confirmation for H. influenzae-real-time MCDA assay. DNA templates from 2 H. influenzae strains and 28 non-H. influenzae strains were tested by the real-time MCDA assay. No reactions were recorded within the non-H. influenzae strains **(A)**. The results were also confirmed by MCDA reactions, indicated by visual detection reagent **(B)** and LFB test **(C)**. **(B)** Tube/Strips 1 and 2 represented two H. influenzae strains, and the other ones represented non-H. influenzae strains. **(C)** TL, test line; CL, control line.

In order to determine the analytic specificity, the real-time MCDA assay was conducted using genomic DNA templates extracted from H. influenzae strains and non-H. influenzae strains (**Table 2**). The positive results were only acquired from the reactions using DNA templates of H. influenzae strains, while all the non-H. influenzae strains were tested to be negative by the real-time MCDA system (**Figure 5**).

### Application of the H. influenzae real-time MCDA assay in clinical specimens

**Figure 6:**
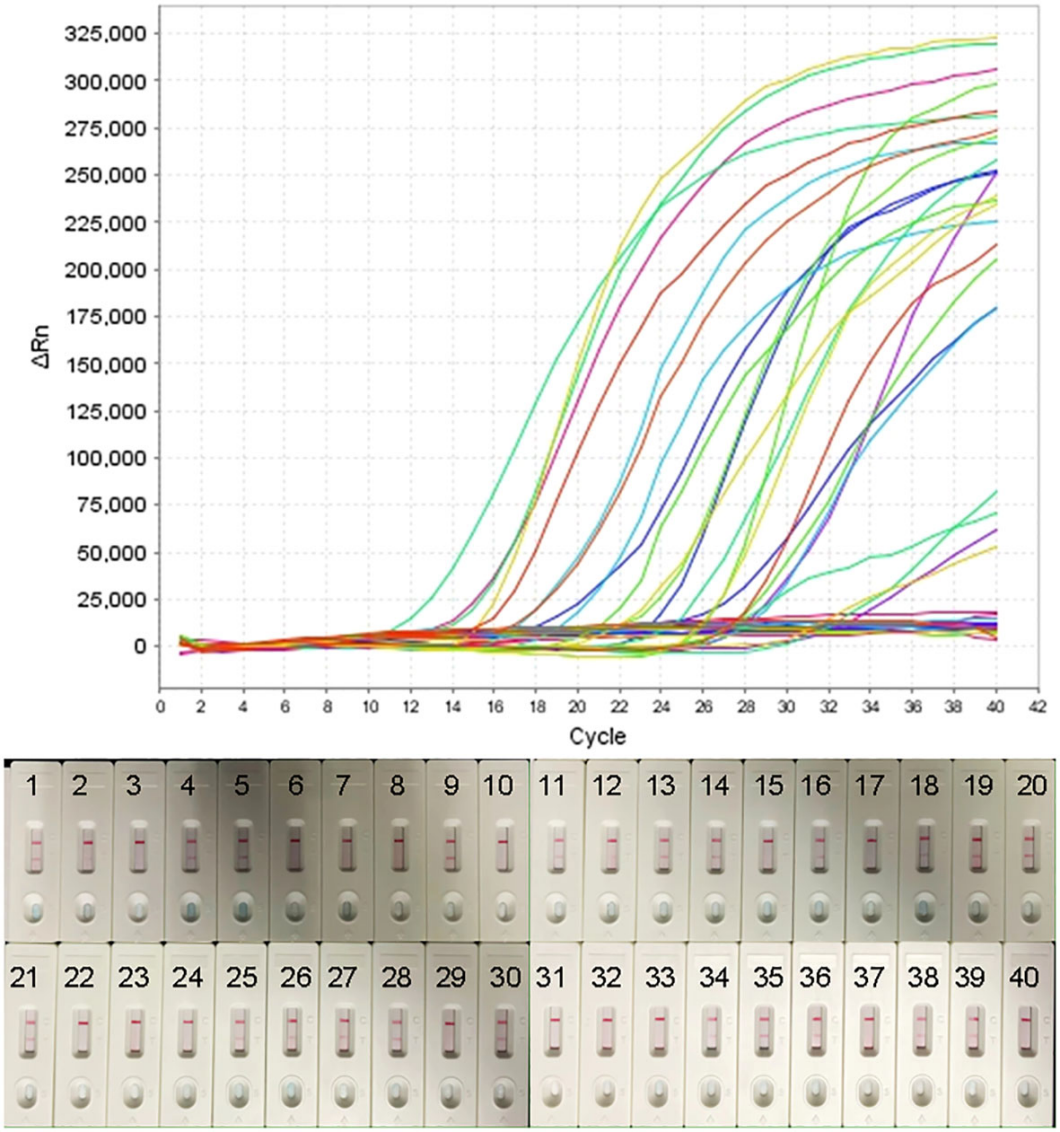
Application of H. influenzae-real-time MCDA assay in clinical specimens. Forty NPS samples were detected by the H. influenzae-real-time MCDA assay and the results showed that 25 samples tested positive. The results were confirmed by the MCDA-LFB test simultaneously.

To further validate the feasibility of the real-time MCDA assay as a method for laboratory diagnosis of H. influenzae, the optimized process was used to detect the retrospectively collected DNA templates extracted from 40 sputum samples, which were previously detected by the LAMP-based assay. The results (**Figure 6**) showed that 25 samples (62.5%) were tested to be positive by the real-time MCDA assay, which was in accordance with that of MCDA-LFB test, while the positive results detected by the LAMP-based assay was 23 (57.5%). The agreement between the real-time MCDA assay and the LAMP test was 95% with a kappa value of 0.896.

## Discussion

As a novel, simple, and rapid isothermal amplification approach for nucleic acid, the MCDA technique has been widely reported for the detection of various pathogens. In previous reports, the MCDA reaction is commonly recommended to be carried out in a thermostatic water bath or heater in consideration of its characteristics of simplicity, convenience, and low cost (Wang et al., 2015; Wang et al., 2016; Wang et al., 2019; Li et al., 2019; Li et al., 2020). The results of the MCDA reaction can be visually judged by chromogenic agent preadded in the reaction vessels or further tested using lateral flow biosensors (Wang et al., 2018; Li et al., 2019; Cao et al., 2021; Wang et al., 2022). The whole process is simple, rapid, efficient, and affordable. However, there is certain subjectivity in the judgment of the results through color change and the possibility of contamination in the further process using biosensors. In addition, the membrane material of the biosensors might have influenced the test results. In this report, we developed a novel real-time MCDA assay, which integrated multiple cross displacement amplification technique and real-time fluorescence examination method. The course of the reaction was real-time monitored and the positive results can be generated in as short as 15 min with no possibility of contamination during the detection stage since the amplification vessels do not need to open.

H. influenzae remains a common cause of illness in children worldwide and account for a considerable proportion of deaths due to bacterial pneumonia (2021; Dong et al., 2020; Fu et al., 2021). Better access to rapid, effective, and reliable laboratory diagnosis is needed especially for definitive clinical diagnosis and timely treatment, which has a positive impact on the course of the infection (Shooraj et al., 2019). In the present study, the highly conserved gene (p6 gene) encoding an outer membrane protein P6 was selected for the identification of H. influenzae. The p6 sequence, found in all strains with or without capsule, was popularly used as the molecular identification marker for H. influenzae in previous studies (Tian et al., 2012; Fan et al., 2018; Albuquerque et al., 2019). Additionally, the sequence has been considered as a very specific region for the diagnosis of H. influenzae (Wang et al., 2022). Here, in the present study, we designed a set of 10 primers targeting the p6 sequence for the real-time MCDA assay. As shown in **Figure 5** and **Table 2**, the primers we used only effectively identified DNA templates from H. influenzae strains under the condition of 63°C, and did not cross-react with the templates from non-H. influenzae bacteria and blank control. The sensitivity of the assay was confirmed using serially diluted genome DNA templates of H. influenzae. As shown in **Figure 4**, the lowest detection level of the real-time MCDA assay was about 10 CFU/reaction, which was more sensitive than that of the LAMP-based assay (500 copies per reaction). In order to evaluate the application of the real-time MCDA assay in laboratory diagnosis of H. influenzae infection, DNA templates extracted from clinical samples were analyzed by the real-time MCDA assay, the MCDA-LFB test, and the LAMP-based assay simultaneously. Among the 40 sputum samples, 25 (62.5%) samples were positive by the real-time MCDA assay and the MCDA-LFB test, and 23 (57.5%) samples were positive by the LAMP assay. The detection rate of the real-time MCDA assay was obviously higher than that of the LAMP test, since the LoD of the LAMP test is 500 CFU per reaction, while the LoD of the real-time MCDA assay determined in the present study is 10 CFU per reaction. Moreover, the results of the real-time MCDA system were real-time reported by the fluorescence quantitative PCR instruments, which was more objective and convenient than the LFB test. Thus, the real-time MCDA assay was more promising in terms of the rapid, sensitive, and specific identification of H. influenzae infection than the MCDA-LFB test, LAMP test, and PCR methods.

In conclusion, the real-time MCDA assay presented in this report, which integrated multiple cross displacement amplification strategy and real-time fluorescence detection technique, displayed much more advantages over the other molecular tests and provided a new strategy for the rapid, efficient, and reliable detection of H. influenzae.

## Data availability statement

The datasets presented in this study can be found in online repositories. The names of the repository/repositories and accession number(s) can be found in the article/supplementary material.

## Ethics statement

The studies involving human participants were reviewed and approved by Ethical Committee of Capital Institute of Pediatrics(Ethical approval number: SHERLLM2019001). Written informed consent to participate in this study was provided by the participants’ legal guardian/next of kin.

## Author contributions

YW designed the study. YW, XC, and DQ supervised this study. CS, XH, NJ, and FX performed the experiments. CS analyzed the data and drafted the manuscript. CS, FX, JF, XH, NJ, YZ, JZ, and ZX contributed to the reagents and materials. YW conducted the software. YW, JZ and XC revised the manuscript. All authors contributed to the article and approved the submitted version.

## Funding

This study was funded by Beijing Nova Program (Grant No. Z211100002121042), the Special Innovation and Promotion Project of Beijing Medical Administration (Grant No. XTCX201820), and the Research Foundation of Capital Institute of Pediatrics (Grant No. PY-2019-07).

## Acknowledgments

We thank Prof. Linqing Zhao (Virus Laboratory of Capital Institute of Pediatrics) and Dr. Wenjian Xu (Bacteriological Room, Clinical Laboratory of Children′s Hospital, Capital Institute of Pediatrics) for their kind help.

## Conflict of interest

The authors declare that the research was conducted in the absence of any commercial or financial relationships that could be construed as a potential conflict of interest.

## Publisher’s note

All claims expressed in this article are solely those of the authors and do not necessarily represent those of their affiliated organizations, or those of the publisher, the editors and the reviewers. Any product that may be evaluated in this article, or claim that may be made by its manufacturer, is not guaranteed or endorsed by the publisher.
